# Cu-Nanoparticle-Doped Amino-MIL-101(Fe)-Functionalized Graphene Oxide Nanocomposite: Synthesis, Characterization, Performance Evaluation and Environmental Applications for Enhanced Tetracycline Antibiotic Removal

**DOI:** 10.3390/nano16090551

**Published:** 2026-04-30

**Authors:** Doaa S. Al-Raimi, Faten M. Ali Zainy, Amr A. Yakout

**Affiliations:** Department of Chemistry, College of Science, University of Jeddah, P.O. Box 80237, Jeddah 21589, Saudi Arabiaaayhussain@uj.edu.sa (A.A.Y.)

**Keywords:** adsorption, Cu-nanoparticles, graphene oxide (GO), metal–organic frameworks (MOFs), tetracyclines, wastewater treatment

## Abstract

Tetracycline antibiotics are increasingly detected in aquatic environments because of their ecological risks and persistence, while conventional wastewater treatment processes are often insufficient for their effective removal from water. Here, we introduce a novel 3D graphene oxide-based nanocomposite that stacks Cu-NPs and amino-functionalized MIL-101(Fe) (denoted by Cu/NH_2_-MIL-101(Fe)@GO) to effectively remove tetracycline (TC) and oxytetracycline (OTC) from environmental water samples. XPS, XRD, TEM, SEM, and FTIR analyses were conducted to characterize the structure and surface morphology of the Cu/NH_2_-MIL-101(Fe)@GO nanocomposite. Overall, it was confirmed that GO, NH_2_-MIL-101(Fe), and Cu-NPs were successfully incorporated, resulting in a porous material with high access to Cu-related sites as well as oxygen- and nitrogen-based functionalities (such as amino-, hydroxy-, and carboxy-groups). This hybrid system facilitates the adsorption by complementary mechanisms like surface complexation/chelation at Cu and Fe centers with the pH-dependent tetracycline species in electrostatic interactions, hydrogen bonding, π–π stacking, and molecule confinement in the metal–organic framework (MOF) pores, and by the synergistic effects at the GO–MOF(Fe)–Cu junction interfaces. The batch adsorption studies showed that the quick and efficient uptake of the two antibiotics at pH 6.5, with removal rates of 99.65–99.83%, was achieved by 15.0 mg of Cu/NH_2_-MIL-101(Fe)@GO at an initial concentration of 20 ppm in 40 min at 25 °C. Equilibrium data were found to be well-fitted by the Langmuir isotherm (*R*^2^ = 0.908–0.909), suggesting monolayer-dominated adsorption with the maximum capacity of 769.8–775.2 mg g^−1^. The adsorption kinetics was well-described by the pseudo-second order model (*R*^2^ = 0.9641–0.9749), which agreed with the strong binding between the tetracyclines and active sites of the nanocomposite. The main novelty of this work consists of the design of a single recoverable platform integrating GO-based preconcentration, pore accessibility of NH_2_-MIL-101(Fe), and Cu-driven complexation, which led to the strong removal of tetracyclines under a relevant range of water conditions. These findings demonstrate that Cu/NH_2_-MIL-101(Fe)@GO could serve as a promising high-efficiency and potentially reusable adsorbent for removing tetracycline from aqueous solution, which provides a more sustainable approach for pharmaceutical wastewater treatment.

## 1. Introduction

Water contamination has intensified with rapid urbanization and industrial growth, creating serious risks for ecosystems and public health and posing a major challenge to sustainable development [1,2]. Among the waterborne contaminants, pharmaceutical residues have received particular attention because they are continuously introduced into aquatic environments, are often not fully removed by conventional treatment processes, and may exert biological effects even at low concentrations.

Antibiotics are widely used in human and veterinary medicine for the treatment and prevention of bacterial infections [3,4,5,6,7,8]. Their extensive use and incomplete removal after consumption have contributed to their widespread occurrence in natural waters and wastewaters as well as to the growing problem of antimicrobial resistance [9]. Among these compounds, tetracycline antibiotics have become an important environmental concern because of their broad-spectrum activity, frequent use, and repeated detection in wastewater effluents and receiving waters [10,11]. Tetracyclines are synthetic or semi-synthetic antibiotics with limited biodegradability, which favors their persistence in aquatic systems.

Because tetracyclines are highly water-soluble, a considerable fraction of the administered dose is excreted unchanged or as active metabolites, allowing these compounds to enter wastewater streams and agricultural runoff [12]. Their presence in the environment can interfere with key microbial processes, including Fe(III) reduction, soil microbial respiration [13], and nitrification [14], and may also affect native microbiota and photosynthetic aquatic organisms [15,16]. In addition to ecological impacts, prolonged exposure to tetracycline residues has been associated with adverse health effects including hepato- and nephrotoxicity, tooth discoloration, impaired bone development, and gastrointestinal disorders [17]. Residues of tetracyclines have also been detected in food products such as milk and fish, while their occurrence in wastewater effluents has led to their recognition as important emerging contaminants. These concerns highlight the need for efficient strategies for tetracycline removal from aqueous environments.

Various treatment processes have been studied for the elimination of TCs such as adsorption [18,19,20], membrane filtration [21], phytoremediation [22], photocatalysis [23], and ozonation [24]. Membrane processes, including nanofiltration and reverse osmosis, provide high separation efficiency with minimal use of chemicals; however, membrane fouling is a critical problem in operation [25]. The consequences of cleaning and fouling mitigation have increased costs and chemical consumption and have shortened membrane lifetimes [26]. In addition, when treating large amounts of dilute antibiotic-containing wastewater, membrane techniques may produce large amounts of retentate for further treatment, which could invalidate advantages when taken to full-scale application.

In view of these difficulties, adsorption is seen as a potentially viable method, particularly with a high-performance adsorbent being now available. Adsorption has the advantage of being simple to operate, requires low energy, and is effective for the removal of a wide range of pollutants [27]. With regard to feasibility, its sustainability depends strongly on adsorbent production and the management of the end of the material’s life; this aspect can be improved through the design of reusable adsorbing materials and easy regeneration methodologies [28]. In some situations, chemical addition is also not required in the adsorption process, which further increases the environmental compatibility [29]. The adsorption performance is dominated by surface chemistry, porosity, and the spatial distribution of functional groups and active sites, which determine the (cross-pH, ionic strength, and co-contaminants affected) affinity and maximum capacity that can be reached under typical environmental water conditions.

Thus, the selection of adsorbing material has a significant impact on the efficiency, cost and feasibility of the adsorption method. Some materials investigated for the removal of TCs are alumina [30], titanium dioxide [31], graphitic nitride with silver nanoparticles [32], seaweed [33], zeolites [34], activated carbon fiber composites [35], and graphene-based adsorbents [36]. Among the various carbon-based sorbents, graphene oxide (GO) has received much attention because its large specific surface area and abundant surface chemistry provide multiple pathways for binding with aromatic, polar, and ionizable contaminants. It possesses hydrophilicity with oxygen-related functionalities such as hydroxyl, epoxide, carboxyl, and carbonyl groups [37], which also promote hydrogen bonding, electrostatic attraction/repulsion, complexation, and π–π stacking with antibiotic molecules. However, the high dispersion stability of GO in water makes its recovery after treatment more challenging, which could add to the operating costs and hinder recyclability. This limitation can be overcome by embedding GO into structured composites such as metal nanoparticles or robust 3D metal–organic frameworks (MOFs) to enhance handling, to minimize leaching, and to allow for fast separation after treatment [38]. In tandem, MOFs offer tunable pore architecture and coordinately open metal centers that may serve as additional adsorption sites, especially for chelating species.

Doping GO with metal atoms is a good way to improve its adsorption properties by creating new active sites and changing the physiochemical properties [39]. Doping may warp the two-dimensional carbon plane and produce defect sites that enhance surface interactions and π–π stacking [40]. Structural modification of GO can alter its surface electronic distribution and create additional adsorption sites, thereby strengthening its interactions with tetracycline molecules. In this context, Cu-containing GO composites are especially attractive because Cu species can coordinate with electron-donating groups present in tetracycline antibiotics, which may enhance adsorption through surface complexation. Previous studies have shown that Cu-modified GO materials can improve the removal of antibiotics and dyes due to changes in surface area, surface charge, and the availability of reactive functional groups within the composite structure [41,42,43]. On this basis, Cu nanoparticles were selected in the present work as a functional dopant to improve the adsorption performance of the GO-based composite.

**Figure 1:**
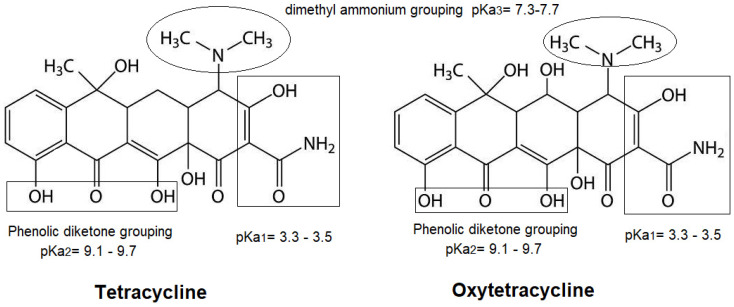
Chemical structures, p*K*_a_ values, and functional grouping of OTC and TC antibiotics.

Herein, we present a novel GO-based nanocomposite with Cu-NPs co-modified by the amino-functionalized iron-based MOF (i.e., NH_2_-MIL-101(Fe)), as a multifunctional adsorbent (Cu/NH_2_-MIL-101(Fe)@GO) for the efficient elimination of tetracycline antibiotics from real water samples. The material is intended to exploit (a) the dispersible, functional group-dense GO core structure; (b) the NH_2_-MIL-101(Fe) porous framework with accessible adsorption sites; and (c) the covalent coordination-capable Cu-NPs to provide a cooperative sorption scenario of synergistic force media. TCs have several electron-donating groups (e.g., diketone, tricarbonyl methane, and dimethylammonium groups) that can be involved in complex formation, while the nanocomposite surface offers the simultaneous presence of electrostatic forces, hydrogen bonds and π–π interactions. The adsorption for tetracycline (TC) and oxytetracycline (OTC) was systematically investigated (Figure 1), and the controlling mechanism was identified by isothermal and kinetic studies and the influence of solution pH and nanocomposite mass. Taken together, this strategy exemplifies a thoughtful composite design for synergistically achieving combined high affinity and feasible recoverability, which may shed light on the efficient removal of tetracycline in real environmental waters.

## 2. Experimental

### 2.1. Reagents

All chemicals were used as received, unless otherwise specified, and were of analytical reagent grade. Copper(II) chloride trihydrate (CuCl_2_·3H_2_O) and sodium hydroxide (NaOH) were used as reagents without further purification. Oxytetracycline (OTC) and tetracycline (TC) were obtained from Genview Chemical Co. Graphene oxide (GO) powder were purchased from Sinopharm Chemical Reagent Co., Ltd. (Shanghai, China). Iron(III) chloride (FeCl_3_), 2-aminoterephthalic acid (NH_2_–H_2_BDC), N,N-dimethylformamide (DMF), acetic acid, and acetonitrile (ACN) were obtained from J&K Scientific (Beijing, China). Solutions of TC and OTC (stock 500 mg L^−1^) were prepared by accurately weighing the amounts and dissolving them in methanol and then stored at 4 °C. Solutions were freshly made each day from working stocks by dilution with Milli-Q water (resistivity > 18.0 MΩ.cm). Figure 1 displays the molecular structures of OTC and TC.

### 2.2. Synthesis of Cu/NH_2_-MIL-101(Fe)@GO Nanocomposite

The Cu/NH_2_-MIL-101(Fe)@GO nanocomposite was prepared in three sequential steps: (i) synthesis of NH_2_-MIL-101(Fe) in the presence of GO nanosheets, (ii) hydrothermal preparation of Cu nanoparticles (Cu-NPs), and (iii) the incorporation of Cu-NPs into NH_2_-MIL-101(Fe)@GO by ball milling.

First, NH_2_-MIL-101(Fe)@GO was synthesized by a solvothermal method using FeCl_3_·6H_2_O as the metal precursor and NH_2_–H_2_BDC as the organic linker [44,45,46]. FeCl_3_·6H_2_O (10.0 mmol, 2.70 g) and NH_2_–H_2_BDC (5.0 mmol, 0.91 g) were dissolved in a mixed solvent of DMF (60 mL) and deionized water (10 mL) under continuous stirring. HCl (37 wt%, 0.5–1.0 mL) was added as a mineral-acid modulator to improve crystallization. A total of 1.5 g of GO was then added to the precursor solution, and the mixture was ultrasonicated for 10 min to ensure good dispersion. The resulting suspension was transferred into a Teflon-lined stainless-steel autoclave and heated at 150 °C for 12 h. After cooling, the solid was collected by centrifugation, washed several times with DMF and ethanol, and then subjected to ethanol solvent exchange to remove residual solvent molecules and unreacted species trapped in the pores. The product was finally dried and activated under vacuum at 120–150 °C to obtain NH_2_-MIL-101(Fe)@GO.

In the second step, Cu-NPs were synthesized hydrothermally in a stainless-steel autoclave at 80 °C for 60 min. CuCl_2_·3H_2_O (1.0 mmol, 0.188 g) was used as the copper precursor, L-ascorbic acid (2 mmol, 0.352 g) as the reducing agent, and the pH was adjusted to 11 using 0.1 *M* NaOH.

Finally, the as-prepared Cu-NPs (0.075 g) were mixed with NH_2_-MIL-101(Fe)@GO (0.50 g) and the mixture was treated in a ball-milling reactor for 30 min at 25 Hz to produce the final Cu/NH_2_-MIL-101(Fe)@GO nanocomposite.

### 2.3. Instrumentation

Surface area and pore size distribution of the textural properties were calculated from N_2_ adsorption–desorption isotherms on a Quantachrome NOVA 4200e instrument (USA). UV determined the concentrations of TC/OTC–Vis spectrophotometry (UVD-3500). SEM (JEOL JSM-6010LV, Tokyo, Japan) and TEM (JEOL JEM-2100V, Tokyo, Japan) were employed to analyze the morphology and microstructure of the Cu/NH_2_-MIL-101(Fe)@GO nanocomposite. Oxidation states and the elemental composition of the surface were investigated by XPS (Thermo ESCALAB 250Xi, Waltham, MA, USA). Crystallinity and phase structure were analyzed with the aid of powder X-ray diffraction (XRD) (Rigaku D/MAX-2550) using Cu Kα radiation. The FTIR spectra (Nicolet 400) were obtained in the range of 4000–400 cm^−1^ to prove the presence of functional groups. Zeta potential analysis was performed on a 1.0 wt% aqueous suspension using a Zetasizer Nano ZS90 (Malvern, UK). The pH of the solution was determined by a calibrated pH meter (Model 810, Waltham, MA, USA; Fisher Scientific, Waltham, MA, USA) with a combined glass electrode.

### 2.4. Adsorption Experiments for OTC and TC

Batch adsorption studies were carried out in centrifuge tubes using Cu/NH_2_-MIL-101(Fe)@GO as the adsorbent. The influence of pH on adsorption was investigated by changing the initial pH of 20 mg. L^−1^ of OTC/TC solution from 2.0 to 11.0 and 15 mg of nanocomposite. To reduce the photodecomposition of tetracyclines, tubes were covered with aluminum foil. The suspensions were sonicated briefly (~2 min), and the pH was regulated by 0.1 mol L^−1^ HCl or NaOH. The blends were shaken on a thermostatic shaker running at 250 rpm for 40 min at 25 °C. At the end of the desired contact time, the solid was filtered, and the supernatant was used to measure the concentration spectrophotometrically at 363 nm with the aid of an external calibration curve prepared from standard TC/OTC solution. To evaluate the impact of nanocomposite mass, different masses (1–50 mg) of the nanocomposite were added to 10 mL of TC/OTC solution (20 mg.L^−1^) at pH 6.5 and 25.0 °C. For the equilibrium isotherm experiments, 10 mg of the nanocomposite was added to 100 mL of TC/OTC solution (10–100 mg L^−1^) at pH 6.5 and 25 °C for 40 min. For the kinetic experiments, 15 mg of adsorbent was added to 10 mL of TC or OTC solution and shaken at 25 °C, and aliquots were withdrawn at selected times between 1 and 60 min.

Removal efficiency and adsorption capacity were calculated using Equations (1) and (2), respectively:

(1) $$\%\;R=\;\frac{C_{o}-\;C_{t}\;}{c_{o}}\;\times \;100$$

(2) $$q_{t}=\frac{{(C}_{o}-C_{t})\;V\;}{m}\;$$

where *C_o_* and *C_t_* (mg L^−1^) are the initial and time-*t* TC/OTC concentrations, *V(L)* is the solution volume, and *m*(g) is the nanocomposite mass. All adsorption tests were conducted three times, and the average values were reported. The precision and accuracy of the method were evaluated through replicate determinations (recovery and RSD). The matrix effects were investigated by comparing the slopes of the matrix-matched calibration curves and those prepared in solvent by one-way ANOVA.

## 3. Results and Discussion

### 3.1. Characterization of Cu/NH_2_-MIL-101(Fe)@GO

**Figure 2:**
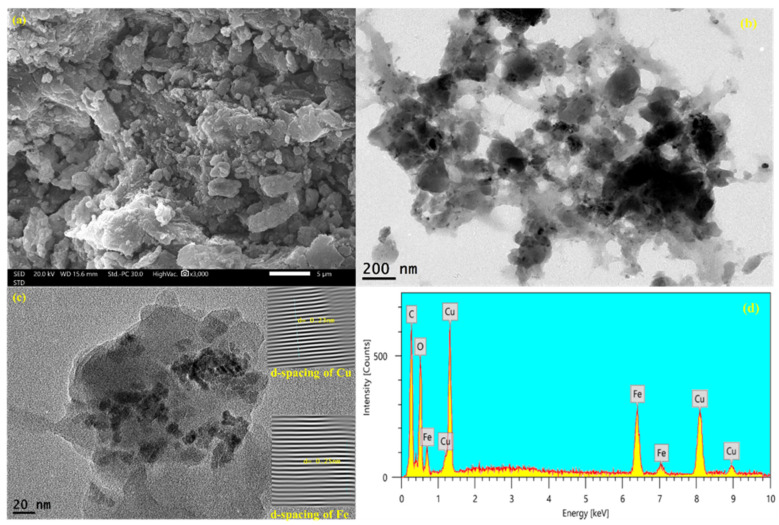
SEM (**a**) and TEM (**b**,**c**) and EDX (**d**) of Cu/NH_2_-MIL-101(Fe)@GO.

The SEM image (Figure 2a) shows a rough, wrinkled, and granular surface, which is consistent with the deposition of NH_2_-MIL-101(Fe) particles on GO sheets. The layered/lamellar structure and large interparticle pores signify the establishment of a hierarchically porous composite instead of a compacted phase, which is advantageous for exposing adsorption/active sites and accelerating mass transfer in the process of tetracycline removal. TEM (Figure 2b) also illustrates a sheet-like GO matrix with darker nanodomains that are assigned to the MOF phase and Cu species, which reflects the uniform stabilization of Cu-containing nanoparticles/clusters within the composite matrix.

In the high-resolution image (Figure 2c), well-defined lattice fringes can be seen with calculated interplanar distances of ~0.23 nm and ~0.25 nm for crystalline domains containing Cu and Fe, respectively, which indicates that both metal-based phases are still crystalline after composite formation and Cu loading. Finally, the EDS spectrum (Figure 2d) shows intense C and O signals (GO framework/oxygenated groups) together with Fe and Cu peaks, confirming the presence of GO, NH_2_-MIL-101(Fe) and Cu in one single nanocomposite. Altogether, this hollow porous nanostructure along with uniform distribution of NPs suggests that such a structure can follow intimate interfacial contact among metal active components as well as porous architecture, which was assumed to contribute to elevated tetracycline removal via the synergistic mechanism of adsorption on functionalized GO/MOF surfaces and metal-mediated interactions.

The ternary nanocomposite showed a specific surface area of 725 m^2^ g^−1^ and a total pore volume of 3.53 cm^3^ g^−1^, indicating a well-developed porous network. The pore-size analysis further showed that most pores were in the mesoporous region, which agrees with the integration of Cu and GO into the MOF structure. Such a hierarchical pore system is favorable for the adsorption of relatively large molecules, as uptake is more likely to take place on the outer surface, near pore openings, and inside accessible mesopores rather than in sterically hindered regions.

**Figure 3:**
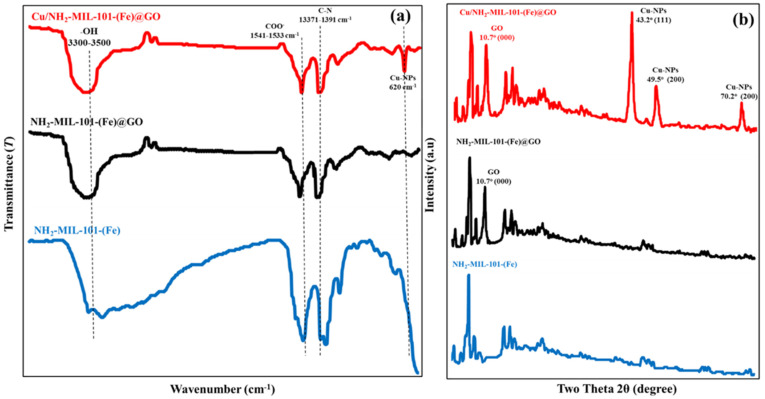
FTIR (**a**) and XRD (**b**) of Cu/NH_2_-MIL-101(Fe)@GO.

The FTIR results of the NH_2_-MIL-101(Fe), Cu/NH_2_-MIL-101(Fe)@GO nanocomposites in the spectra wave numbers (cm^−1^) (Figure 3a) clearly reveal the functional groups responsible for the adsorption. The broad band at 3300–3500 cm^−1^ in the case of the pristine NH_2_-MIL-101(Fe) can be credited to O–H and N–H stretches of the coordinated water vapor and amino groups, respectively. The band at 1541–1533 cm^−1^ is specific for the asymmetric stretching of the coordinated carboxylate (COO^−^) groups of the organic linker, which confirms that the MIL-101 framework is intact. When GO and Cu species are introduced, the band intensities are changed significantly with some slight shifts and a new band located at about 620 cm^−1^ for Cu–O vibrations, which suggests the Cu-NPs have been successfully anchored in the composite matrix [47]. After adsorption of TC and OTC, in the O–H/N–H stretching region and the carboxylate related-bands, distinct changes can also be found, indicating that they are involved in the binding. The weakening and slight shifting of these peaks also confirm the participation of the surface hydroxyl groups, amino groups, and metal–oxygen sites in the bonding with antibiotic molecules. These spectral transformations are consistent with a cooperative adsorption mechanism in which hydrogen bonding between TC/OTC functional groups and Fe–O/Cu–O active sites, coordination interactions, and potential π–π stacking with the graphitic domains of GO are involved. Notably, the retention of main framework vibrations indicates maintenance of the structural integrity of the Cu/NH_2_-MIL-101(Fe)@GO nanocomposite after adsorption, which reflects the stability of the Cu/NH_2_-MIL-101(Fe)@GO nanocomposite and its potentially practical application for antibiotic removal.

The XRD analysis of the GO, NH_2_-MIL-101(Fe), and Cu/NH_2_-MIL-101(Fe)@GO nanocomposite clearly confirms the successful synthesis of the core–shell structure as well as the retention of crystallinity after the functionalization process (Figure 3b). Clearly, the characteristic diffraction peak of GO at 2θ ≈ 10.7° related to the (001) plane can be seen, signifying the existence of oxygenated graphene layers. Pure NH_2_-MIL-101(Fe) shows its characteristic crystalline reflections in the low angle region, which umbrellas the well-reported structure of the MIL-101 scaffold, thus indicating the preparation of a well-defined porous structure [48,49,50,51,52]. After the addition of Cu-NPs, three more intense peaks tagged with 2θ ≈ 43.2°, 49.5°, and 70.2° are indexed as (111), (200), and (220) planes of face-centered cubic metallic Cu separately. These peaks, along with peaks related to GO and NH_2_-MIL-101(Fe) confirm the successful growth of crystalline Cu-NPs over the surface without collapsing the pristine MOF. After adsorption of TC and OTC, no additional crystalline impurity phase was observed, and the main diffraction peaks were not significantly changed, indicating that the adsorption process is surface interaction and coordination interaction rather than a structural transformation. The preserved crystallinity demonstrates the high structural stability and reusability of the Cu/NH_2_-MIL-101(Fe)@GO nanocomposite in the field of pollutant removal.

**Figure 4:**
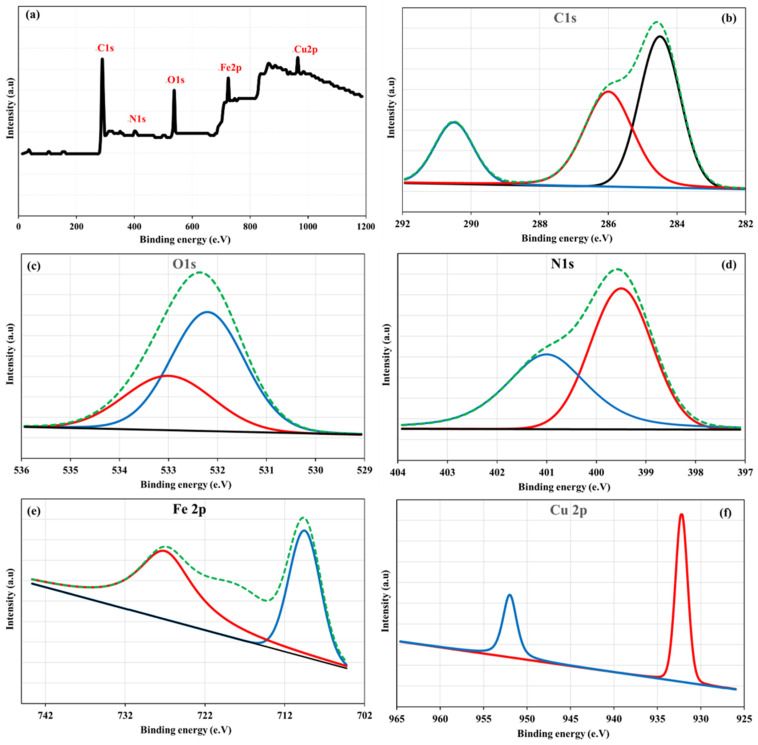
Full survey (**a**) and elemental (**b**–**f**) XPS of Cu/NH_2_-MIL-101(Fe)@GO.

The XPS results strongly indicate the successful preparation of Cu/NH_2_-MIL-101(Fe)@GO and detail the surface-specific interactions in both TC and OTC adsorption. The survey spectrum showed the existence of C, N, O, Fe, and Cu elements, which was in line with the content of the composite (Figure 4a). In the high-resolution C1s spectrum (Figure 4b), the major component at ~284.6 eV is attributed to C–C/C=C bonds in the aromatic framework and in the GO sheets, the peaks at ~286 eV and ~288–289 eV are assigned to C–N/C–O and O–C=O groups, respectively. After TC and OTC adsorption, small shifts or variations in intensity in these oxygen- and nitrogen-based carbon species imply that they may be involved in the binding interactions [47,48,49,50,51,52]. The O1s spectrum is deconvoluted to be centered at ~531–533 eV, associated with metal–oxygen (Fe–O/Cu–O) and surface hydroxyl or carbonyl groups, and reveals a significant change after adsorption, suggesting a coordination between antibiotic molecules and metal active sites (Figure 4c). The N1s profile is (Figure 4d) near ~399–401 eV, which can be attributed to the –NH_2_/–NH– group of the MOF linker; the shift to higher binding energy observed after TC/OTC sorption suggests that amino groups are implicated either by hydrogen bonding or by repulsion. In the region of Fe 2p spectrum (Figure 4e), the typical Fe 2p_3_/_2_ and Fe 2p_1_/_2_ doublet representing Fe^3+^ in NH_2_-MIL-101 is maintained after loading; however, slight variation in peak shape and position after adsorption suggests the formation of a surface complex. Likewise, the Cu 2p spectrum exhibits (Figure 4f) the characteristic signals of Cu^2+^ with no sign of phase transformation, suggesting that Cu species serve as stable coordination centers. Taken together, these spectral changes indicate that the removal of TC and OTC is attributed to a synergistic mechanism, which includes surface complexation with Fe-O, Cu-O sites, and hydrogen bonding with –NH_2_ and oxygenated functionalities as well as π–π interactions with graphitic domains of GO, without causing the collapse of the nanocomposite.

### 3.2. Impact of Solution pH and Nanocomposite Mass

**Figure 5:**
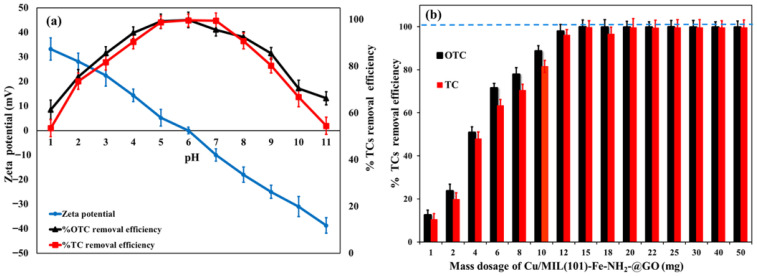
The impact of zeta potential and pH on the adsorptive removal of OTC and TC (**a**) and mass nanocomposite impact (**b**) of the Cu/NH_2_-MIL-101(Fe)@GO nanocomposite at 25.0 °C and 40 min contact time.

Solution pH was the main factor controlling the adsorption of TC and OTC by the Cu/NH_2_-MIL-101(Fe)@GO, because it affects both the surface properties of the nanocomposite and the ionization state of the antibiotic molecules. It can be seen that as the zeta potential decreases, the zeta potential also tends to be more positive with increasing pH, and is nearly neutral at around pH ≈ 6.0; there is a change in the sign of the dominant surface charge at the surface, as shown in Figure 5a. In agreement with this trend, the removal rate dramatically increased throughout the acidic range and obtained the highest value at the weak acid or neutral condition (≈pH 6–7), with a removal over 99.65–99.83% within 40 min and declined rapidly in alkaline media. This pH-dependent behavior fits well with the multi-p*K*_a_ nature of TC/OTC: the molecules at pH can be present mainly as cationic species in strongly acidic solutions or as zwitterions at neutral to faintly acidic pH as well as anionic species at alkaline pH. At lower pH, adsorption is favored by strong noncovalent interactions such as π–π interactions between aromatic tetracyclic rings of TC/OTC and π-rich domains of GO that also participate in cation–π interactions between the protonated dimethylammonium group of TC/OTC and the aromatic electron cloud of GO, and these interactions could be enhanced at lower pH [15,18,41].

In the medium pH range (approximately 3–9), uptake is strengthened by additional binding to metal-containing sites: hydrated Cu/Fe surface species (e.g., CuOOH/FeOOH-like moieties) can act as Lewis’s acid/base centers, whereas Cu-NPs offer supplementary coordination/chelation options by means of surface interaction with oxygen- and nitrogen-based functional groups of TC/OTC. The combined electrostatic and specific interactions explain the strong adsorption efficiency at the region close to the point of zero charge. In contrast, the surface of the composite becomes more negatively charged, and the species of TC/OTC move toward anionic at high pH (generally >9); the resulting electrostatic repulsion, competing with OH^−^, in conjunction with inhibitions of cation–π/π–π interactions by deprotonation, is responsible for the significant reduction in removal [15,18].

Nanocomposite mass effect was in line with the site availability concept (Figure 5b). In the suitable pH range, increment of the Cu/NH_2_-MIL-101(Fe)@GO nanocomposite mass (1–50 mg in 10 mL of 20 mg·L^−1^ TC or OTC) led to a drastic enhancement in the removal efficiency because more adsorption/coordination sites and larger areas could be accessed. For instance, the removal was raised from ~20% to ~98% as the nanocomposite mass increased from 2 to 12 mg and continued to increase more gradually up to ~15 mg. Above this value, the efficiencies of removal tended toward a limit, suggesting that the limit to adsorption was the concentration of solute and not the availability of vacant sites; also, at high TEMs, particle agglomeration and overlapping of active sites can reduce the benefit increment per unit mass added. Hence, 15 mg was chosen as the working TEM for the rest of the studies since it offered near-quantitative removal with good material economics.

### 3.3. The Impact of Time and Adsorption Kinetics

**Figure 6:**
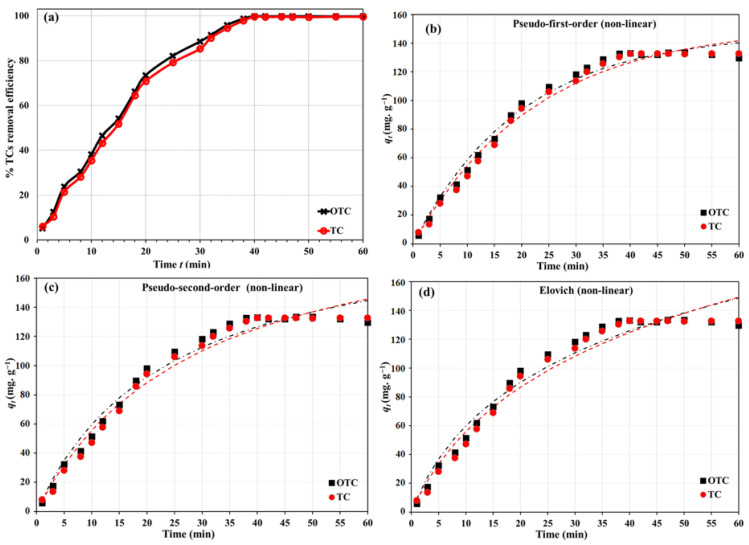
Impact of contact time (**a**), nonlinear kinetic equations of PSO (**b**), PFO (**c**), and Elovich (**d**) models for the adsorptive removal of OTC and TC by the Cu/NH_2_-MIL-101(Fe)@GO nanocomposite at 25.0 °C, 20 mg L^−1^ as the initial concentration, pH 6.5, and 15 mg of nanocomposite.

To better interpret the adsorption kinetics, the experimental data were fitted using the pseudo-first-order (PFO), pseudo-second-order (PSO), and Elovich models (Figure 6b–d) [53,54,55,56]. The adsorption of TC and OTC was rapid during the first 20 min, followed by a slower approach to equilibrium, and near-complete removal was achieved within approximately 40 min under the selected working conditions. The experimental time profiles were analyzed using the nonlinear forms of the PFO, PSO, and Elovich models. The fitted curves are shown in Figure 6b–d, and the resulting parameters are summarized in Table 1.

For TC and OTC, the nonlinear PFO model provided the best overall description of the experimental data. It yielded the lowest RMSE values (0.498 mg g^−1^ for OTC and 0.504 mg g^−1^ for TC) and the highest *R*^2^ values (0.9863 for OTC and 0.9865 for TC) among the three tested models. The fitted equilibrium capacities were 14.72 mg g^−1^ for OTC and 15.22 mg g^−1^ for TC, with corresponding *k*_1_ values of 0.0507 and 0.0445 min^−1^, respectively. The higher apparent *k*_1_ value for OTC reflects faster uptake under identical experimental conditions, which may be associated with subtle differences in molecular structure, accessibility of binding sites, and interaction strength with the nanocomposite surface. These results indicate that the overall uptake profiles are well-described by a rapid occupation of the available sites followed by a gradual asymptotic approach to equilibrium.

The nonlinear PSO model also represented the data reasonably well, but its fit quality was inferior to that of the PFO model. The RMSE values increased to 0.664 mg g^−1^ for OTC and 0.644 mg g^−1^ for TC, while the fitted *q_e_* values (20.25 and 21.58 mg g^−1^, respectively) were clearly higher than the experimental plateau uptake. This behavior suggests that when fitted in nonlinear form over the full-time domain, the PSO model tends to overpredict the terminal capacity for the present system.

The Elovich model showed the largest deviations from the experimental profiles (RMSE = 0.808 mg g^−1^ for OTC and 0.767 mg g^−1^ for TC), although its acceptable overall fit still supports a heterogeneous adsorbent surface and a progressive decrease in adsorption rate with increasing surface coverage. The fitted Elovich parameters were α = 0.999 mg g^−1^ min^−1^ and β = 0.158 g mg^−1^ for OTC, and α = 0.850 mg g^−1^ min^−1^ and β = 0.141 g mg^−1^ for TC.

Overall, the nonlinear treatment demonstrates that the kinetic model ranking differs from that obtained by linearization. Under optimum conditions, the adsorption of OTC and TC on Cu/NH_2_-MIL-101(Fe)@GO is better represented by the nonlinear PFO model than by the nonlinear PSO or Elovich equations. The kinetic results are consistent with the structure of Cu/NH_2_-MIL-101(Fe)@GO, where the high surface area, mesoporous framework, and coexistence of GO oxygenated groups, NH_2_-MIL-101(Fe) functionalities, and Cu/Fe coordination sites provide a large number of accessible and energetically non-equivalent adsorption sites, leading to rapid initial uptake and heterogeneous surface adsorption behavior.

### 3.4. Sorption Isotherms

For the equilibrium isotherm study, 10 mg of Cu/NH_2_-MIL-101(Fe)@GO was equilibrated with 100 mL of TC or OTC solution at initial concentrations of 10–100 mg L^−1^ at pH 6.5 and 25 °C for 40 min, and the resulting data were used to evaluate the Langmuir and Freundlich adsorption models. The adsorption uptake in both cases sharply increased within the range of 5–20 mg L^−1^ of *C_e_*, then slowly leveled off with a further increase in the adsorbate concentration (OTC: *q*_*e*_ = 491–497 mg g^−1^; TC: *q*_*e*_ = 477–481 mg g^−1^) at *C*_*e*_ = 40–50 mg L^−1^. This saturation behavior is similar to what can be observed during the rapid occupation of numerous high-energy sites at low concentration and the subsequent incremental occupation of low-energy or more distant ones as the concentration or driving force increases, leading to constant removal efficiency at high *C_e_* [57]. To understand the adsorption process, the experimental results were modeled using the Langmuir and Freundlich isotherms, as shown in Table 2. A slightly better overall fit was observed for the Langmuir model (monolayer adsorption on a limited number of sites [57]) in both antibiotics, with determination coefficients of *R*^2^ = 0.908 for OTC and *R*^2^ = 0.909 for TC. The corresponding Langmuir capacities were high, with *q*_max_ = 775.2 mg g^−1^(OTC) and 769.8 mg g^−1^ (TC), whereas the constants of affinity were *K*_*L*_ = 0.0437 and 0.0399 L mg^−1^ for the OTC and TC, correspondingly. The *K*_*L*_ values were relatively high, indicative of a strong nanosorbent–adsorbate interaction, which is probably the main reason behind the sharp sorption at low *C*_*e*_ and the fast attainment of almost full uptake at high solute concentrations. Most notably, the plateau capacities observed experimentally (~500 mg g^−1^) suggest that most of the easily accessible sites were saturated within the investigated window and hence, the higher *q*_max_ values are model-based extrapolations that take into account the large theoretically achievable site density of the composite rather than an achievable capacity for the considered concentration range.

The Freundlich model [58], which considers heterogeneous surfaces and has nonuniform energies of adsorption, also fitted well (OTC: *R*^2^ = 0.888; TC: *R*^2^ = 0.899), revealing that the Cu/NH_2_-MIL-101(Fe)@GO surface was not completely homogeneous (which is expected for a hybrid material where MOF domains are sandwiched between sheets of GO). The Freundlich parameters further confirm that adsorption is favorable with values of *n* = 1.54 and 1.51 for OTC and TC (i.e., *n* > 1), and quite impressive capacity coefficients of *K*_*F*_ = 47.73 and 43.52 for OTC and TC, respectively. These *n* values tell us that adsorption is thermodynamically favorable over the entire range studied and that adsorption is becoming increasingly less incremental as coverage increases, which is consistent with the observed behavior (i.e., a transition from a high-slope region at low *C_e_* to a saturation region at high *C_e_*).

The equilibrium data suggest that the adsorption of OTC and TC on Cu/NH_2_-MIL-101(Fe)@GO does not follow a single idealized adsorption model. Instead, the observed behavior is consistent with the combined influence of surface heterogeneity and progressive site occupation. Under the studied conditions (pH 6.5), both OTC and TC are present in mixed ionic forms, which enables multiple types of interaction with the nanocomposite surface. These interactions likely include electrostatic attraction between charged antibiotic species and surface functional groups, hydrogen bonding with oxygen- and nitrogen-containing sites, π–π interactions with the graphitic domains of GO, and coordination or surface complexation with accessible Cu- and Fe-containing sites in the composite. Therefore, the adsorption process is more appropriately described as a multimodal interaction mechanism on a heterogeneous surface rather than as purely monolayer adsorption on a uniform set of sites.

The marginally greater capacities and affinity for OTC when compared to TC (a higher *q*_*e*_ plateau and *K_L_*) are in accordance with a stronger overall binding of OTC under the conditions considered, which supports the superior removal performance at increasing concentration. Different adsorbents/nanosorbents reported in the literature for TC removal are summarized in Table 3. The nanosorbents are clays, carbon-based materials, magnetic composites, functionalized GO, and MOFs. Meanwhile, different interaction processes, including van der Waals and electrostatic interactions, are mostly presented. The maximum adsorption capacities of the Cu/NH_2_-MIL-101(Fe)@GO nanocomposite for TC were compared with those of reported adsorbent materials [59,60,61,62,63,64,65,66,67,68,69,70] and analyzed in comparison with other sorbents.

Functionalization of the GO backbone with NH_2_-MIL-101(Fe) and Cu-NPs leads to a novel nanocomposite that has a lot of merits. Cu-NPs also contribute to the stabilization of GO sheets, avoiding their restacking and enabling better TC adsorption through electrostatic and metal-complexation interactions. NH_2_-MIL-101(Fe) can effectively bind to different ionized species of tetracyclines (TCH_3_^+^, TCH_2_, TCH^−^, TC^2−^) via electrostatic forces and H-bonding with solution pH and can improve the adsorption performance of Cu/NH_2_-MIL-101(Fe)@GO. GO functions in TC adsorption in two ways: the GO facilitates π–π stacking. Cu/NH_2_-MIL-101(Fe)@GO also demonstrated a great synergetic effect among all of the components, dramatically promoting performance in TC removal.

### 3.5. Contribution of Cu-NPs and NH_2_-MIL-101(Fe) to Adsorption Performance

**Figure 7:**
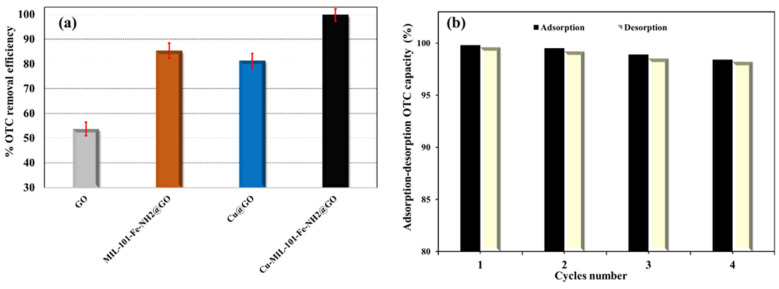
The impact of the Cu-NPs and NH_2_-MIL-101(Fe) nanoparticles on the Cu/NH_2_-MIL-101(Fe)@GO nanocomposite’s OTC adsorptive removal efficiency (**a**) as well as its reusability (**b**).

To elucidate the individual and synergistic roles of Cu-NPs and NH_2_-MIL-101(Fe) in the designed Cu/NH_2_-MIL-101(Fe)@GO adsorbent, comparative adsorption experiments were performed at pH 6.5 using pristine GO, Cu@GO, NH_2_-MIL-101(Fe)@GO, and Cu/NH_2_-MIL-101(Fe)@GO. The results (Figure 7a) clearly show that integrating both Cu and the Fe-MOF markedly enhanced the OTC uptake relative to GO alone or single-component hybrids.

The enhancement effect of Cu-NPs can be explained by two synergistic mechanisms. (i) Cu-NPs improve the restacking/aggregation of GO layers and then contribute to the retention of accessible surface area and a higher concentration of exposed adsorption sites. Secondly, Cu exhibits chemically active sites that facilitate the specific binding of OTC via surface complexation in this pH range, which is in line with the high metal affinity of tetracycline-dressed molecules. Under aqueous conditions, Cu oxides may undergo partial hydrolysis yielding hydrated species (e.g., CuOOH-like species), which act as amphoteric surface moieties and further assist surface complexation with diverse tetracycline species under various pH regimes. At the same time, the NH_2_-MIL-101(Fe) provides Fe-based coordination sites, including open/partially accessible metal sites and metal–oxo/hydroxo groups that can be involved in ligand exchange and inner-sphere complexation, forming Fe–O-OTC bonds. The diffusion of OTC into the porous framework is also possible, allowing access to the MOF cages, and there the adsorption is again enhanced by the confinement effect and by several weak host–guest interactions along with coordination with Fe-associated sites. Notably, the –NH_2_ functionalization makes the surface more polar and provides more interaction sites (such as hydrogen bonding and dipole interactions), which may lead to stronger binding to the tetracyclines than that of the nonfunctionalized ones.

Taken together, these results indicate that the dual incorporation of Cu-NPs and NH_2_-MIL-101(Fe) into a GO matrix results in a synergistic adsorption platform, which significantly enhances the removal of OTC.

### 3.6. Reusability and Structural Stability of Cu/NH_2_-MIL-101(Fe)@GO

The stability of Cu/NH_2_-MIL-101(Fe)@GO was evaluated under consecutive adsorption–desorption–regeneration cycles. Desorption of OTC was performed with a 0.20 mol L^−1^ formic acid–methanol solution at a short contacting time of 3.0 min. The regenerated sorbent was collected, dried, and weighed again after each cycle. After six regeneration cycles, the material surprisingly showed no mass loss, which is an indication of ideal mechanical integrity and resistance to leach.

At the same time, the adsorption capacity of the recycled nanocomposite was much closer to that of the fresh one. As can be seen from Figure 7b, the removal efficiency of OTC slightly dropped from 99.83% for the first run to 96.51% for the fourth run, indicating that Cu/NH_2_-MIL-101(Fe)@GO could be recycled with the efficiency loss being no more than two runs at least. The stability of the structure was confirmed by powder XRD (Figure S1): the pattern of the nanocomposite after OTC adsorption was almost identical to that of the original sample, indicating that the crystal framework was maintained during the adsorption/desorption processes. The sustained OTC removal efficiency over repeated adsorption–desorption cycles, together with the absence of measurable mass loss and the preservation of the main XRD features, indicates that Cu/NH_2_-MIL-101(Fe)@GO maintains good structural stability and reusability under the tested conditions for the removal of the tetracycline-class antibiotic.

### 3.7. Cu/NH_2_-MIL-101(Fe)@GO Nanocomposite’s Mechanism

TC and OTC antibiotics have several ionizable and electron-donating groups (phenolic–enolic groups, amide and hydroxyl groups, and a dimethylammonium group), which give rise to their interaction with heterogeneous adsorbent materials by simultaneous chemical and physicochemical mechanisms. Based on the XRD changes observed after adsorption, the pH-dependent adsorption behavior, and the comparative performance of the component nanocomposite, the overall adsorption of TC/OTC on Cu/NH_2_-MIL-101(Fe)@GO is anticipated as a synergistic effect of electrostatic attraction/repulsion, surface complexation with metal centers, hydrogen bonding, π–π interactions, and pore-filling/partitioning in the MOF layers. The proportion of each process is highly dependent on the pH of the solution, the ionic strength, and the surface charge distribution over the composite.

*(i)*  *First interaction: Electrostatic interactions*

TCs are amphoteric and have cationic, zwitterionic, and anionic forms at different pH values. At the same time, the surface of the composite is composed of negatively charged oxygenated groups of GO (–COO^−^/–O^−^), protonated amine groups of NH_2_-MIL-101(Fe) (–NH_2_/–NH_3_^+^), and pH-dependent surface charges of metal-based sites. Protonation of the dimethylamino group of tetracycline and the partial protonation of MOF amino groups can favor attractive forces with negatively charged GO moieties under acidic conditions, and multipoint binding occurs at or about neutral pH in the presence of zwitterionic tetracycline and mixed-charge surface groups. At alkaline pH, the deprotonation of tetracycline and GO escalates, and electrostatic repulsion between both species can be established but may be offset by divalent metal complexation and H-bonding; both interactions may be enhanced as tetracycline deprotonates and becomes a stronger chelator.

*(ii)*  *Second interaction: Coordination/chelation with Cu and Fe centers*

Inner-sphere complexation with nonspecific binding sites on the surface of exposed metal sites constitutes a relevant pathway for tetracycline sorption. Tetracyclines are known to be effective chelators because of their vicinal oxygen donors (especially the β-diketone/phenolic–enolic system), which allows for bidentate or multidentate coordination to metal centers. In this hybrid material, Cu-NPs offer coordinately active surface atoms, which can bind with the deprotonated oxygen donors and thus facilitate the generation of surface Cu–TC complexes. Meanwhile, NH_2_-MIL-101(Fe) provides an Fe-based coordination environment (open/partially accessible metal sites and metal–oxo/hydroxo species) that can serve as a complexation/ligand exchange site to establish an Fe–O(TC/OTC) framework. Metal–ligand interactions of this kind are often stronger than non-specific electrostatic interactions and could account for fast adsorption and enhanced stability in a system where electrostatic interactions alone tend to break down. Hence, the presence of Cu- and Fe-based binding sites will facilitate “multisite” complex formation, where tetracycline is anchored to different regions of the composite through different functional moieties.

*(iii)*  *Third interaction: Hydrogen bonding and acid–base-assisted binding*

GO is decorated with hydroxyl, epoxide, carbonyl, and carboxyl groups, and –NH_2_ groups are grafted on the surface of NH_2_-MIL-101(Fe), which may serve as hydrogen-bond donors or acceptors according to its protonation state. Tetracyclines have numerous hydroxyl and amide functional groups that can participate in hydrogen bonding. As a result, strong H-bond interactions can occur between TC/OTC and (i) GO oxygenated functional groups, (ii) amino-functional groups on the MOF linkers, and (iii) metal-coordinated –OH/–H_2_O functionalities. These interactions contribute to improved adsorption at neutral pH and may result in susceptibility effects due to potential orientation of the antibiotic close to the metal binding sites, thus favoring subsequent chelation. Such local acid–base interactions (such as between NH_2_-MIL-101(Fe) protonated –NH_3_ + and tetracycline deprotonated oxygen donors) can in principle also lead to the stabilization of adsorbed species via ion pairing in conjunction with H-bonding.

*(iv)*  *Fourth interaction: π–π stacking and hydrophobic/van der Waals contributions.*

TCs have fused aromatic rings that can be stacked with the sp^2^ carbon domains of GO through π–π stacking. This is the more relevant interaction when electrostatic repulsion is turned off (e.g., around the zwitterionic forms), and with antibiotics being adsorbed parallel to the graphenic planes. Besides π–π stacking, non-specific van der Waals forces and hydrophobic partitioning into more reduced graphitic domains may also enhance the overall affinity, particularly in complex aqueous systems due to the fact that competing ions may partially mask the electrostatic forces.

*(v)*  *Fifth interaction: Pore-filling and confinement within NH_2_-MIL-101(Fe)*

NH_2_-MIL-101 type frameworks provide large cages as well as accessible porosity within them that can favor adsorption by means of pore-filling and confinement effects. TC/OTC molecules can diffuse into the pores of the MOF and become entrapped therein through steric confinement, multiple weak interactions with the pore walls, and coordination to Fe-associated sites. The amino functionalization (–NH_2_) increases polarity and introduces additional specific sites for interaction that may enhance affinity toward tetracyclines in comparison with unfunctionalized counterparts. The MOF domain is thus not only a high-surface area host, but also a microenvironment that localizes tetracyclines at reactive metal sites and functional groups.

*(vi)*  *Synergistic interfacial effects and role of GO support*

GO plays a dual role: (i) providing additional adsorption sites and π-rich domains, and (ii) acting as a dispersive scaffold that limits the aggregation of MOF particles and Cu-NPs, increasing the accessibility of binding sites. Interfacial coupling between GO and NH_2_-MIL-101(Fe) can create heterogeneous junctions where charge distribution and local polarity differ from either component alone, generating “hot spots” for adsorption. Likewise, anchoring Cu-NPs onto GO/MOF interfaces can increase the fraction of exposed Cu surface atoms and promote cooperative binding, where tetracycline first accumulates on GO or within MOF pores and then forms stronger metal complexes with nearby Cu or Fe sites. This sequential capture-preconcentration followed by complexation can rationalize both rapid kinetics and high uptake capacity.

The unique character of the present adsorbent lies in the deliberate assembly of three synergistic adsorption domains, oxygenated GO, amino-functionalized Fe-MOF, and Cu-NPs surfaces, into one recoverable nanocomposite. In contrast to single-phase sorbents, which predominantly exploit either π–π interactions (carbon materials) or pore-filling (porous frameworks), this composite structure allows for multimodal interactions: fast preconcentration on GO, entrapment and site-dense adsorption in NH_2_-MIL-101(Fe), and strong inner-sphere chelation on Cu/Fe clusters. The simultaneous presence of Cu and Fe coordination spheres is especially favorable for tetracyclines, as it facilitates the formation of stable, multidentate metal–ligand complexes over a wide pH range, and the GO substrate ensures a superior dispersion and accessibility of active sites. Such a cooperative process may serve as a rational explanation for the faster and more robust tetracycline removal in environmentally relevant waters, which also suggests the potential in designing composite interfaces that combine “capture” (GO/MOF) with “locking” (metal complexation) functionalities in one material platform.

### 3.8. Performance of Cu/NH_2_-MIL-101(Fe)@GO for OTC Removal from Environmental Water Samples

The Cu/NH_2_-MIL-101(Fe)@GO-based SPE method for tetracyclines (TC and OTC) was evaluated by recovery, precision, and calibration linearity for its applicability. The slopes of the calibration curves obtained from the matrix-matched standards were statistically similar to those obtained in solvent (*p* > 0.05), as determined by one-way ANOVA, suggesting effective suppression of matrix effects and the possibility of using external calibration without significant bias. The method was also validated with typical real waters (river, tap, and commercial bottled water) to illustrate its usefulness. As shown in Table 4, the trace/background level of all unspiked samples was either very low in the case of the river water sample (1.0–3.0 × 10^−2^ mg L^−1^), or not detected (ND) in both the tap water and distilled water samples, which suggested that there was a slight native contamination and a low measurement limit was allowed for adequate quantification. Upon the three levels of spiking (~5, 10 and 15 mg L^−1^), the nanocomposite showed excellent recoveries in all matrices, ranging from 99.6 to 100.0% with good repeatability (RSD 2.2–4.2%). Remarkably, such excellent extraction efficiency was achieved despite the wide range of sample pH (≈5.81–8.22), demonstrating the resistance of the extraction/adsorption process to real water chemistries. Taken together, good near-quantitative recoveries and low variance demonstrate that Cu/NH_2_-MIL-101(Fe)@GO can provide an accurate, reproducible, and matrix-insensitive platform for the detection and elimination of tetracycline-class antibiotic in complex water samples.

## 4. Conclusions

Herein, the design and application of a 3D GO-based nanocomposite comprising Cu-NPs with amino-functionalized MIL-101(Fe); (Cu/NH_2_-MIL-101(Fe)@GO) as an efficient adsorbent for tetracycline antibiotics in aqueous media are presented. Full characterization confirmed the synthesis of a porous, hierarchically structured material wherein GO acts as a functionalized scaffold, NH_2_-MIL-101(Fe) as accessible adsorption sites and closed pores, and Cu-NPs as superior metal-active centers. The nanocomposite structure enabled the fast and efficient adsorption of tetracycline-class drugs with 99.64–99.83% removal at pH 6.5 by using as low as 15 mg of adsorbent at 20 ppm within 40 min at 25 °C, thus notably by the natural pH of the solution, pointing out practical application with no or only the slightest chemical modification.

The Langmuir model, theoretical maximum adsorption capacity, and pseudo-second-order kinetics could properly describe the adsorption behavior, suggesting that there were strong and site-specific interactions between tetracycline and the surface of the composites. Mechanistically, a synergistic multimodal binding mechanism (related to electrostatic interactions dependent on pH-tetracycline speciation, hydrogen bonding between tetracycline and oxygenated and amino groups, π–π interactions between tetracycline and GO domains, pore-filling/confinement within the NH_2_-MIL-101(Fe) cages, but in particular coordination/chelation at Cu and Fe sites that stabilizes adsorbed tetracycline species via surface or inner-sphere complex formation) is responsible for the high removal efficiency. The synergy of these two mechanisms permits rapid and high adsorption capacity in environmentally relevant media. Reuse experiments demonstrated the efficient stability of Cu/NH_2_-MIL-101(Fe)@GO. The nanocomposite retained efficient activity after four adsorption–desorption cycles, and 0.20 mol L^−1^ formic acid–methanol solution regeneration sufficiently restored the adsorption sites, in accordance with reversible binding contributions in addition to strong complexation. In addition to excellent efficiency, the material also displays practical advantages in post-treatment processing and separation because its composite nature and diminished portioning versus pristine GO offer help in recuperation and cyclical use. Overall, the main novelty of this work is the design of a dual-function, query-recoverable platform based on the integration of GO-based preconcentration and π-rich domains with the porous and amino-functional properties of NH_2_-MIL-101(Fe), and at the same time, using Cu-NPs to add coordination-active sites for “locking” tetracycline by metal–antibiotic complexation. This synergistic GO–MOF–Cu design provides a stable and effective way to treat tetracycline-contaminated environmental waters and represents a general approach to trap other chelating pharmaceutical residues by customized, multifunctional MOF/GO/metal nanocomposites.

## Figures and Tables

**Table 1:** Nonlinear kinetic equations for the adsorption of OTC and TC on the Cu/NH_2_-MIL-101(Fe)@GO nanocomposite at 25.0 °C, pH 6.5, and 15 mg of nanocomposite [53,54,55,56].

Kinetic Model	Nonlinear Kinetic Equation	Calculated Parameters	OTC	TC
*Sorption Kinetic Equations*				
PFO	$q_{t}=q_{e}\;\cdot \;(1-e^{-k_{1}t})$	*q_e_* (mg·g^−1^)	114.72	115.22
		*k*_1_ (min^−1^)	0.5070	0.4451
		*R* ^2^	0.9863	0.9865
		*RMSE* (mg·g^−1^)	0.4985	0.5040
PSO	$q_{t}=\;\frac{k_{2}q_{e}^{2}\;t}{{1+k}_{2}q_{e}\;t}$	*q_e_* (mg·g^−1^)	200.25	221.58
		*k*_2_ (g mg^−1^·min^−1^)	2.1 × 10^−2^	1.6 × 10^−2^
		*R* ^2^	0.9756	0.9779
		*RMSE* (mg·g^−1^)	0.6645	0.6444
Elovich	$q_{t}=\frac{1}{\beta }\;\ln\;(1+\alpha \beta \;t)\;$	*α* (mg·g^−1^·min^−1^)	0.9993	0.1582
		*β* (g·mg^−1^)	0.8504	0.1409
		*R* ^2^	0.9639	0.9688
		*RMSE* (mg·g^−1^)	0.8083	0.7666

*q_e_* and *q_t_* (mg·g^−1^) are the adsorption capacity at equilibrium and at time *t*, respectively. *C_e_* is the TC/OTC at equilibrium. *k*_1_ and *k*_2_ are the first- and second-order adsorption rate constants of the kinetic models. *α* (mg·g^−1^·min^−1^) refers to the initial adsorption rate. *β* (g·mg^−1^) is related to surface coverage and activation energy.

**Table 2:** Fitting parameters of the adsorption isotherm models for TC and OTC adsorption onto Cu/NH_2_-MIL-101(Fe)@GO at pH 6.5, using 10.0 mg of nanocomposite in 100 mL of solution, after 40 min of contact time at 25.0 °C.

Adsorption Isotherm Model/ Linear Equation	Isotherm Parameters	OTC	TC
Langmuir isotherm			
$\frac{1}{q_{e}}\;=\;\frac{1}{q_{m}}\;+\;\frac{1}{K_{L}q_{m}C_{e}}$	*q_m_* (mg·g^−1^)	775.2	769.8
	*K_L_* (L·mg^−1^)	0.0437	0.0399
	*R* ^2^	0.9075	0.9095
Freundlich isotherm			
$\ln q_{e}\;=\;\ln K_{F}\;+\;\frac{1}{n}\;\ln C_{e}$	*K_F_* (L·mg^−1^)	47.73	43.52
	*n*	1.536	1.507
	*R* ^2^	0.8877	0.8992

**Table 3:** OTC and TC maximum adsorption capacity (*q_m_*) values by the Cu/NH_2_-MIL-101(Fe)@GO nanocomposite in comparison to different adsorbents.

Adsorbent	*q_m_* (mg g^−1^)	Reference
TC		
Activated sludge	72	[59]
Montmorillonite	84	[60]
Fe_3_O_4_–rGO composites	95	[61]
Palygorskite	99	[62]
Iron-enriched biochar	99.5	[63]
Multi-walled carbon nanotubes	100	[64]
Rectorite	140	[65]
MIL-68(Al)/GO	228	[66]
GO	313	[67]
Single-walled carbon nanotubes	340	[64]
Al(III)-based MOF CYCU-3	428.1	[68]
Smectite	462	[69]
Cu/MnO_2_/CS/GO nanocomposite	479.2	[70]
Cu/NH_2_-MIL-101(Fe)@GO nanocomposite	769.8	The present work
OTC		
Bamboo-derived biochar	21.5	[71]
Montmorillonite	34	[60]
Coconut shell (loaded nano zero valent iron)	167.9	[72]
Multi-walled carbon nanotubes	190	[64]
GO	212	[67]
Clay	800	[72]
Cu/MnO_2_/CS/GO nanocomposite	526.3	[70]
Cu/NH_2_-MIL-101(Fe)@GO nanocomposite	775.2	The present work

**Table 4:** Adsorptive removal performance of OTC from spiked environmental water matrices using the Cu/NH_2_-MIL-101(Fe)@GO nanocomposite.

Sample	pH	Spiked Concentration (mg L^−1^)	Detected Concentration (mg L^−1^)	% Recovery ^a^ ± RSD ^b^
River water	5.81	5.1	0.02	99.6 ± 3.6
		10.0	0.01	99.9 ± 4.2
		15.0	0.02	99.9 ± 2.5
Tap water	7.75	5.2	0.01	99.8 ± 2.7
		10.1	0.02	99.8 ± 3.1
		15.2	0.03	99.8 ± 2.8
Bottled water	8.22	5.1	ND	100.0 ± 3.5
		10.0	0.01	99.9 ± 3.2
		15.0	ND	100.0 ± 2.2

^a^ Recovery %. ^b^ Relative standard deviation (*n* = 3).

## Data Availability

The original contributions presented in this study are included in the article/Supplementary Materials. Further inquiries can be directed to the corresponding author.

## Supplementary Materials

The following supporting information can be downloaded at: https://www.mdpi.com/article/10.3390/nano16090551/s1, Figure S1: XRD spectra of Cu/NH_2_-MIL-101(Fe)@GO before and after OTC adsorption.
