# Supplementary figures and images for: Cu-Nanoparticle-Doped Amino-MIL-101(Fe)-Functionalized Graphene Oxide Nanocomposite: Synthesis, Characterization, Performance Evaluation and Environmental Applications for Enhanced Tetracycline Antibiotic Removal
Source: Nanomaterials (Basel). 2026 Apr 30;16(9):551. doi: 10.3390/nano16090551 (PMC13164626; doi:10.3390/nano16090551)

Figure S1: XRD spectra of Cu/NH<sub>2</sub>-MIL-101(Fe)@GO before and after OTC adsorption.

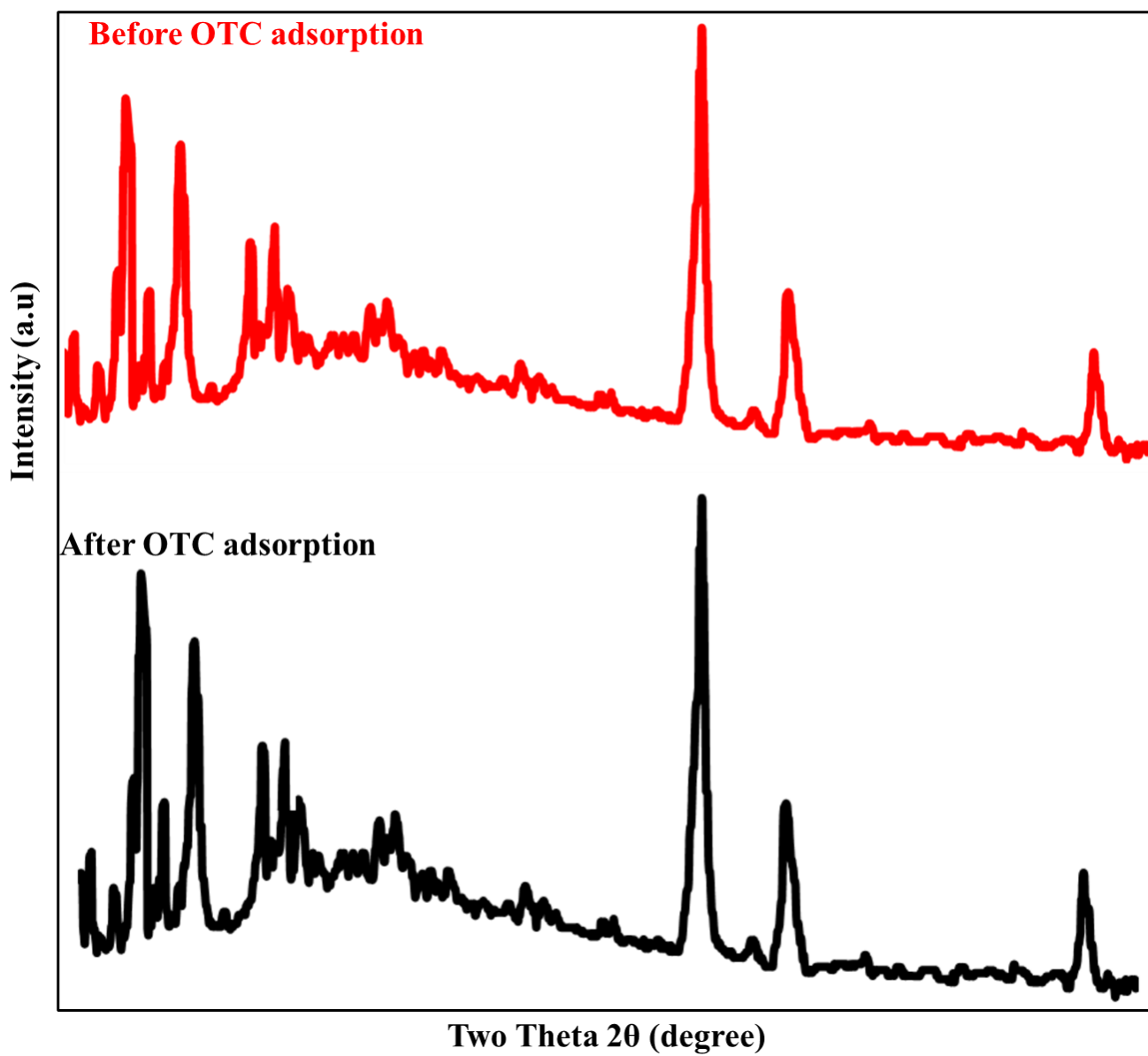

Supplement: Supplementary file 1 [file nanomaterials-16-00551-s001.zip › nanomaterials-4243479-supplementary.pdf]
